# Radiological follow-up strategies in adolescent idiopathic scoliosis patients: A best evidence synthesis by systematic review

**DOI:** 10.1016/j.bas.2025.105865

**Published:** 2025-11-05

**Authors:** Jules Cool, Anne Mareille Post, Barend J. van Royen, Mario Maas, Geert J. Streekstra, Faridi S. Jamaludin, Joost van Schuppen

**Affiliations:** a Amsterdam UMC, Location AMC, Department of Orthopaedic Surgery and Sports Medicine, Meibergdreef 9, Amsterdam, the Netherlands; b Amsterdam UMC, Location AMC, Emma Children's Hospital, Meibergdreef 9, Amsterdam, the Netherlands; c Amsterdam UMC, Location AMC, Department of Radiology and Nuclear Medicine, Meibergdreef 9, Amsterdam, the Netherlands; d Amsterdam UMC, Location AMC, Biomedical Engineering and Physics, Meibergdreef 9, Amsterdam, the Netherlands; e Amsterdam UMC, Location AMC, Medical Library, Meibergdreef 9, Amsterdam, the Netherlands; f Amsterdam Movement Sciences, Musculoskeletal Health - Restoration and Development, Amsterdam, the Netherlands

**Keywords:** Adolescent idiopathic scoliosis, Imaging, Radiology, Follow-up, Curve progression, Systematic review

## Abstract

**Introduction:**

Patients with adolescent idiopathic scoliosis (AIS) undergo frequent radiographic evaluations due to the progressive nature of the deformity. However, current intervals between radiographs are based on expert consensus, rather than scientific evidence.

**Research question:**

To determine appropriate radiological follow-up intervals based on estimated monthly deformity changes in observed AIS patients.

**Material and methods:**

A systematic search was conducted in MEDLINE, EMBASE and the Cochrane Library from inception until January 8th, 2025. Inclusion criteria were: (1) studies including AIS patients observed using radiological follow-up alone, and (2) reporting average monthly curve progression or curve progression rate over a pre-determined follow-up interval. Methodological quality was assessed using the Quality in Prognostic Studies (QUIPS) tool. Deformity progression rates were defined to categorize patient- and radiological characteristics into low-, moderate-, or high-grade progression with corresponding recommendations for radiological follow-up.

**Results:**

Nine eligible studies were identified, including a total of 2326 AIS patients. Monthly curve progression was sufficiently reported for four prognostic factors. Most articles reported curve progression rates according to patients' bone age, dictating a six-month radiological follow-up interval until skeletal maturity is approached. Nearly all studies showed a high risk of bias along with heterogeneity in the prognostic factors analyzed for curve progression. Thus, data regarding the suggested radiological follow-up intervals was inconclusive.

**Discussion and conclusion:**

Due to the high risk of bias and heterogeneity among the included studies, conclusive evidence could not be established. Limited evidence suggests radiographic follow-up every six months until skeletal maturity is approached, with annual follow-up thereafter.

## Highlights

• Evidence-based consensus is needed on radiological follow-up in observed AIS.
• Curve progression in growing AIS patients depends on multiple prognostic factors.
• Six-month X-ray follow-up until skeletal maturity is advised by best-evidence synthesis.

## 1 Introduction

Adolescent Idiopathic Scoliosis (AIS) is the most common type of scoliosis, with a prevalence of 4 % reported by the Scoliosis Research Society (SRS) (Scoliosis Research Society). Girls of adolescent age are primarily affected, with female-to-male ratios of up to 10:1 among patients with a more extensive deformity (more than 30°) (Konieczny et al., 2013). The diagnosis is primarily stated by a Cobb angle exceeding 10° on standing posterior-anterior (PA) full-spine radiographs, as also defined by the SRS (Kim et al., 2010; Negrini et al., 2018). Depending on the size of the curve, either observation, brace treatment or surgical correction of the deformity is indicated (Negrini et al., 2022; Karimi and Rabczuk, 2018). The conservative treatment options include scoliosis-specific exercise (SSE) or observation only (Karimi and Rabczuk, 2018). For the observation group, follow-up consultation is solely based on the overtime monitoring of the deformity using standing PA full-spine radiographs, enabling timely interventions in case of progression of the deformity (Kim et al., 2010; Knott et al., 2014). Observed AIS patients, therefore commonly undergo long-lasting clinical follow-up (Cheung and Cheung, 2021). Most growth, or peak height velocity, can be expected before the menarche (Dimeglio and Canavese, 2013). These patients must be closely monitored for curve progression, while patients who are near skeletal maturity can be subjected to fewer evaluations. An important predictor of remaining patient growth and curvature progression therefore is the bone age, which can be used to select and plan treatment possibilities as brace therapy or (growth-sparing) fusion (Yamamoto et al., 2020). Multiple other predictive factors have been identified, including the initial magnitude of the deformity, curve type and gender (Janicki and Alman, 2007).

Due to the progressive nature of AIS, regular full-spine radiological evaluations are necessary to monitor curve progression during observation. This often results in a high number of full-spine radiographs conducted over the total follow-up period, and subsequent cumulative exposure to ionizing radiation (Cool et al., 2023). This cumulative ionizing radiation exposure is disadvantageous in generally young AIS patients with a subsequent vulnerability to the carcinogenic effect of radiation, especially due to the onset of breast growth and high proportion of red bone marrow present in spine, sternum and pelvis (Blebea et al., 2007; Doody et al., 2000). Currently, there exists a lack of protocols outlining time intervals for imaging studies in the follow-up of patients after the diagnosis of AIS. The 2016 SOSORT (The International Society on Scoliosis Orthopaedic and Rehabilitation Treatment) consensus paper recommended intervals ranging from 12 to 24 months for radiological follow-up in the treatment of idiopathic scoliosis, depending on age and the Risser stadium of the patient (Negrini et al., 2018). These intervals were also adopted by other international societies including the American College of Radiology (ACR), as described in the ACR Appropriateness Criteria® (American College of Radiology, 2018). However, the recommended intervals were based on consensus between experts, but not on evidence from the literature. Such evidence-based recommendations are necessary to limit the cumulative ionizing radiation exposure of AIS patients during clinical visits while preserving diagnostic and treatment quality including timely recognition of deformity progression. Therefore, the aims of this systematic review are as follows: (1) to estimate the monthly changes in the scoliotic curve in AIS patients; (2) to identify the factors that influence the progression of scoliosis in AIS patients, and (3) to state evidence-based recommendations regarding radiological follow-up intervals, based on aforementioned overtime progression rates.

## 2 Material and methods

This systematic review is written according to the Preferred Reporting Items for Systematic Reviews and Meta-Analyses (PRISMA) criteria (Page et al., 2021). Prior to the start of this systematic review, the protocol was registered on PROSPERO (CRD42024513095).

### 2.1 Inclusion criteria and literature search

A literature search was conducted by a medical information specialist (FSJ) from inception until January 8th, 2025, in three online databases (MEDLINE (Ovid), EMBASE (Ovid), Cochrane Library (Wiley)). The following terms were used in the initial search: Scoliosis, adolescent, curve progression and follow-up studies. The full search is listed in the supplementary material (appendix A). Articles were considered eligible when reporting an average curve progression (Cobb angle increase) over a given period (for example degrees/month). Articles written in English, French, German or Dutch were included. No requirements were set for the year of publication. Follow-up studies (both prospective and retrospective) were considered eligible study designs. Only studies including skeletally immature AIS patients, treated using radiological observation were incorporated. For interventional studies, only the results from an observed control group were taken into account. Studies that did not report deformity progression for specific prognostic factors (for example bone age or curve type), were excluded at full-text screening since they presented overall progression data which does not allow for specific regular follow-up recommendations. Backwards citation searching was conducted to incorporate potentially eligible articles that were not identified in the initial search.

### 2.2 Report selection

Titles and abstracts were retrieved from the articles that resulted from the search and duplicates were removed. Two reviewers (JC & AMP) independently screened all titles and abstracts using the pre-determined inclusion criteria. Thereafter, selected studies from the title and abstract screening were assessed on full-text eligibility. Discrepancies between the two authors at both title and abstract-, and full-text screening were discussed. If no consensus could be obtained, a third reviewer (JVS) made the final decision.

### 2.3 Data extraction

The primary and secondary outcome measures were extracted from the included articles by one author (JC) using a pre-determined data-extraction form. In accordance with the objective of this systematic review, over-time curve progression (e.g., average over-time increase in Cobb angle) was used as the primary outcome measure. Patients and related prognostic factors for curve progression were categorized according to the expected time it takes to reach a threshold of 5° curve progression, a commonly accepted definition of treatment failure (Faldini et al., 2022). Appropriate recommendations for radiological follow-up intervals were subsequently stated. The risk groups were defined as described in Table 1: low-grade (curve-progression of <5° per year, with a subsequent recommendation for annual radiological follow-up) moderate-grade (curve-progression of 5° in 6–12 months, with a subsequent recommendation for radiological follow-up every 6 months), and high-grade (curve-progression of >5° in 6 months, with a subsequent recommendation for radiological follow-up every 3–4 months).

**Table 1:** Risk classification and corresponding recommendations for radiological follow-up intervals.

Risk category	Time until a 5° in curve progression is anticipated	Suggested interval for radiological follow-up
Low-risk of curve progression	>12 months	Every 12 months
Moderate-risk of curve progression	6–12 months	Every 6 months
High-risk of curve progression	<6 months	Every 3–4 months

The following study characteristics were extracted.

• Study design
• Number of observed patients included
• Male/female ratio of observed patients
• Mean patient age at presentation and end of follow-up
• Mean Cobb angle at presentation and end of follow-up

### 2.4 Risk of bias assessment

Two authors (JC & AMP) independently assessed the selected studies on quality and risk of bias using the Quality in Prognostic Studies (QUIPS) tool. This tool assesses the risk of bias in six domains (study participation, study attrition, prognostic factor measurement, outcome measurement, study confounding, statistical analysis and reporting) as also elaborated on by Hayden et al. (2006). Each domain is rated as low, moderate or high risk of bias, and an overall risk of bias is given based on the risk of bias found within all six domains. As the QUIPS tool does not provide a singular overall rating that summarizes the risk of bias, the overall risk of bias was noted according to Grooten et al. (2019). If all six domains scored a low risk of bias, or a maximum of one domain as moderate risk of bias, the study was categorized as low risk of bias. A high risk of bias was noted for studies scoring a moderate risk of bias for three or more domains, or if one domain was scored as high risk of bias. A moderate risk of bias was applicable for all studies in between. Especially aspects within the included studies relevant to the intended objective of the current systematic review (for example conversion to brace treatment during observation, and other patient characteristics that could influence over-time curve progression such as age and Cobb angle at first visit) were taken into account during the risk of bias assessment.

### 2.5 Analysis

Based on the deformity progression rate described for patient subgroups in the included studies (skeletal age, curve type etc.), recommendations for radiological follow-up intervals are described. The level of evidence for each recommendation was determined based on the updated guidelines by Furlan et al. (2009).

• Strong evidence: consistent (>75 %) findings in multiple (≥2) high-quality studies
• Moderate evidence: findings in one high-quality study and consistent findings (>75 %) in multiple (≥2) low-quality studies
• Limited evidence: findings in one high-quality study and consistent findings (>75 %) in ≥3 low-quality studies
• Inconclusive evidence: findings found in <3 low-quality studies
• Conflicting evidence: <75 % of the studies reported consistent findings

## 3 Results

### 3.1 Studies included

**Fig. 1:**
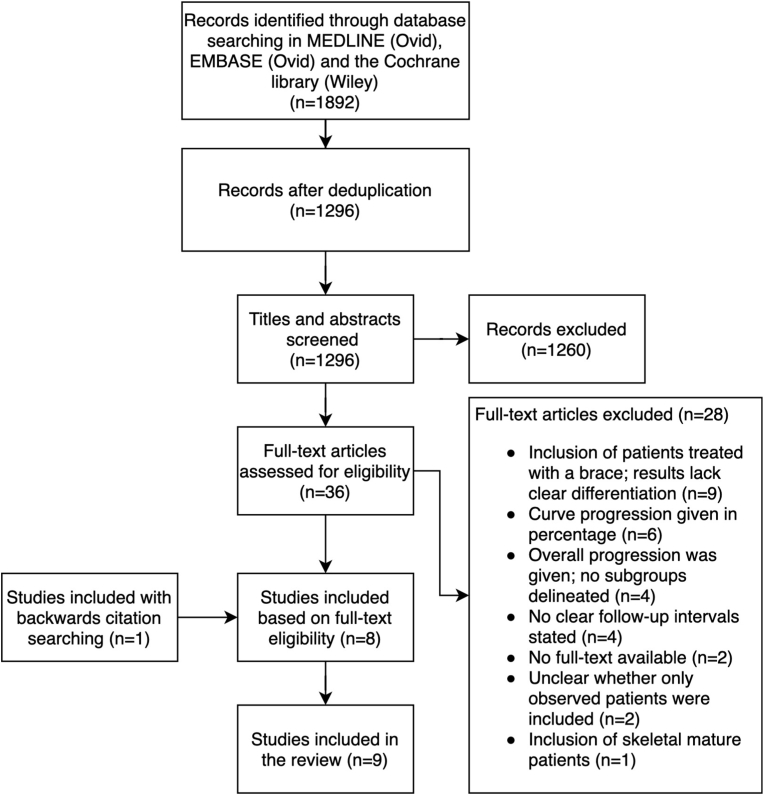
PRISMA flow diagram of abstract and full-text screening.

A total of 1296 articles resulted from the initial literature search after deduplication. Title and abstract screening resulted in 36 possibly eligible articles. Eight articles were included after full-text screening (Cheung and Cheung, 2021; Cheung et al., 2018; Escalada et al., 2005; Gao et al., 2021; Hori et al., 2024; Schleder et al., 2023; Ylikoski, 2005; Zapata et al., 2019). Reasons for exclusion are noted in the PRISMA diagram (Fig. 1). One additional article was identified through backward citation searching (Duval-Beaupere, 1996). In the end, a total of nine studies were included after the literature review.

### 3.2 Methodological quality

Two reviewers determined the risk of bias according to the QUIPS tool (Table 2). All nine studies were identified as having a high risk of bias. Five studies converted patients from observation to brace treatment when curve progression was detected, which resulted in shortcomings within the study attrition domain. Within these studies, data regarding the progression of the deformity was used until the initiation of brace treatment (Cheung and Cheung, 2021; Cheung et al., 2018; Escalada et al., 2005; Hori et al., 2024; Ylikoski, 2005). Three studies included a small population of observed AIS patients and were therefore classified as having a high risk of bias (Gao et al., 2021; Schleder et al., 2023; Zapata et al., 2019). The remaining study was considered as high risk primarily due to shortcomings within the confounding domain (Duval-Beaupere, 1996).

**Table 2:**
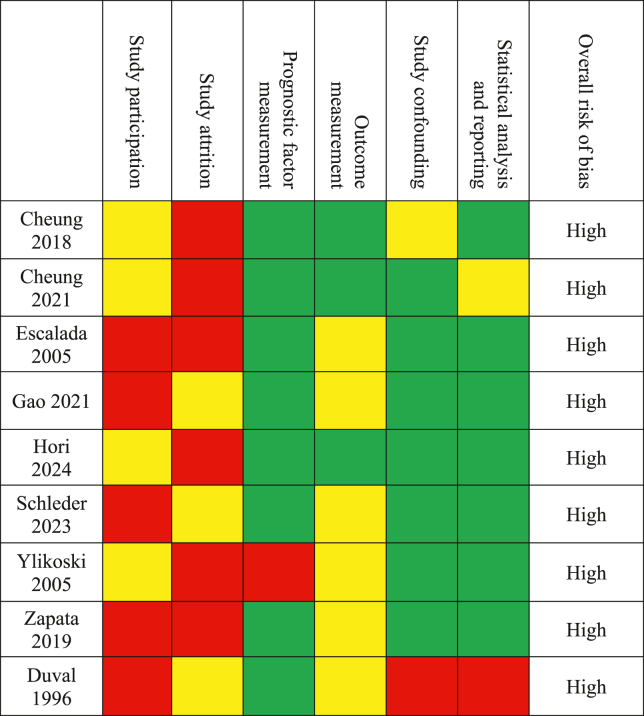
Results of the risk of bias assessment.

### 3.3 Study characteristics

The baseline characteristics of the included studies are listed in Table 3. The included studies were published between 1996 and 2024. A total of 2326 AIS patients were included, with the treatment plan comprising radiological observation alone. The sample size of observed AIS patients within the individual studies ranged between 8 and 878 patients. Six studies reported mean age at initial appointment (T0), with a pooled mean (pooled for sample size) of 13.31 ± 0.64 (range 12.00–15.80) years (Cheung and Cheung, 2021; Cheung et al., 2018; Gao et al., 2021; Schleder et al., 2023; Ylikoski, 2005; Duval-Beaupere, 1996). A pooled mean of 17.33 ± 0.55 (range 16.30–17.62) year was reported at the end of follow-up, which was noted by two studies (Schleder et al., 2023; Zapata et al., 2019). The mean Cobb angle at T0 was reported by seven studies, with a pooled mean of 22.87 ± 4.30 (range 16.90–57.90) degrees (Cheung and Cheung, 2021; Cheung et al., 2018; Gao et al., 2021; Hori et al., 2024; Schleder et al., 2023; Ylikoski, 2005; Duval-Beaupere, 1996). The Cobb angle at final follow-up was reported by four studies, for which a pooled mean of 28.18 ± 18.46 (range 18.70–69.79) degrees was applicable (Gao et al., 2021; Hori et al., 2024; Schleder et al., 2023; Zapata et al., 2019).

**Table 3:** Study characteristics.

First author, year of publication	Study design	Patients (n)	Males (n)	Patient population	measurement of curve progression	Subgroups	Age at T0	Age at end of follow-up	Cobb angle at T0 (°)	Cobb angle at end of follow-up (°)
Cheung et al., 2018	Retrospective	318	82	Risser stage 0–3, less than 2 years post-menarche	Mean progression rate, °/month	DRU grade	12.0 ± 1.5 years	–	21.6 ± 4.8	–
Cheung et al., 2021	Retrospective	878	230	Untreated AIS	Mean progression rate, °/month	DRU grade Risser stage Sanders stage Gender	13.3 ± 1.5 years	–	23.0 ± 6.0	–
Escalada et al., 2005	Retrospective	88	0	Girls, curve magnitude >10°	Mean progression rate, °/6 months	Years pre/post menarche	–	–	–	–
Gao et al., 2021	Retrospective	21	4	10–17 years, Risser stage ≥3, Cobb angle 20–40°.	Mean progression rate over total follow-up period (33.1 ± 6.2 months)	Risser stage ≥3	15.8 ± 1.5 years	–	27.8 ± 4.1	28.1 ± 5.2
Hori et al., 2024	Retrospective	123	–	SMS 3A or SMS 3B	Mean progression rate, °/month	SMS 3A	–	–	16.9 ± 7.5	18.7 ± 10.6
						SMS 3B	–	–	17.5 ± 6.1	19.1 ± 8.7
Schleder et al., 2023	Retrospective	29	5	AIS patients awaiting surgical treatment (10–18 years)	Mean progression rate, °/year	Lenke classification	13.34 ± 2.62 years^a^	17.62 ± 3.33 years	57.9 ± 14.67 (range 40–101)	69.79 ± 13.79 (range 50–106)
Ylikoski, 2005	Retrospective	535	0	Girls, untreated AIS	Mean progression rate, °/year	Curve type Growth velocity	13.9 (range 9–19) years	–	23.4 (range 5–60)	–
Zapata et al., 2019	Prospective	8	0	10–17 years, Risser stage 0, Cobb angle 12–20°	Mean progression rate, °/6 months	Risser stage 0	–	16.3 ± 3.1 years	–	20.1 ± 7.1
Duval-Beaupere, 1996	Retrospective	326	5	Cobb angle <30°	Mean progression rate, °/year	Curve type	161.9 ± 24.5 months	–	21.6 ± 14.5	–

a Age at the indication for surgical correction of the deformity.

### 3.4 Study results

Three studies noted the average progression of the deformity in the Cobb angle per year (Schleder et al., 2023; Ylikoski, 2005; Duval-Beaupere, 1996), three studies in the Cobb angle per month (Cheung and Cheung, 2021; Cheung et al., 2018; Hori et al., 2024), and two studies in the Cobb angle per six months (Escalada et al., 2005; Zapata et al., 2019). One study reported the Cobb angle progression over the total treatment period (33.1 ± 6.2 (range 25–41) months) (Gao et al., 2021). Calculated in terms of monthly progression and noted for each subgroup analyzed for curve progression, monthly curve progression ranged from 0.0009° to 2.87°, for AIS in the whole group.

A large heterogeneity was observed in patient- and radiological prognostic factors analyzed for curve progression within each individual study. Curve progression was described for skeletal age in five studies (Cheung and Cheung, 2021; Cheung et al., 2018; Gao et al., 2021; Hori et al., 2024; Zapata et al., 2019), with three studies reporting the Risser stage (Cheung and Cheung, 2021; Gao et al., 2021; Zapata et al., 2019), two studies the DRU (Distal Radius and Ulna) classification (Cheung and Cheung, 2021; Cheung et al., 2018) and two studies the Sanders maturation scale (Cheung and Cheung, 2021; Hori et al., 2024). Curve progression was analyzed for curve type in three studies (Schleder et al., 2023; Ylikoski, 2005; Duval-Beaupere, 1996), with two studies reporting the curve progression according to curve pattern (Ylikoski, 2005; Duval-Beaupere, 1996), and one study reporting curve progression for the Lenke classification (Schleder et al., 2023). One study analyzed how AIS progressed in the years leading up to, or following menarche (Escalada et al., 2005), and one study analyzed curve progression related to growth velocity (Ylikoski, 2005).

### 3.5 Best-evidence synthesis

Risk factors, and subsequent proposals for radiological follow-up intervals, were categorized for each patient- and radiological prognostic factor analyzed for curve progression (Table 4). All studies were scored as having a high risk of bias, and there was a large heterogeneity present in prognostic factors analyzed for curve progression. As a result, the data is inconclusive regarding the use of the suggested radiological follow-up intervals and corresponding patient- and surgical characteristics in clinical practice.

**Table 4:** Risk-classification of patient and radiological characteristics with corresponding recommendations for radiological follow-up.

	Factors corresponding with a low-risk of progression (≥ annual follow-up)	Factors corresponding with a moderate-risk of progression (follow-up every 6 months)	Factors corresponding with a high-risk of progression (follow-up every 3–4 months)
***Bone age***			
Cheung et al., 2018			
- DRU classification	Radius grade 8, 9, 10, 11	4, 5, 6, 7	–
	Ulnar grade 3, 7, 8, 9	2, 4, 6	5

Cheung et al., 2021			
Girls			
- Risser classification	Risser stage 2, 3, 4, 4+, 5	0, 0+, 1	–
- DRU classification	Radius grade 8, 9, 10, 11	5, 6, 7	–
	Ulnar grade 7, 8, 9	4, 5, 6	–
- Sanders classification	Sanders stage 1, 5, 6, 7, 8	2, 3, 4	–
Boys			
- Risser classification	Risser stage 1, 3, 4, 4+, 5	0, 0+, 2	–
- DRU classification	Radius grade 8, 9, 10, 11	5, 6, 7	–
	Ulnar grade 4, 7, 8, 9	3, 5, 6	–
- Sanders classification	Sanders stage 6, 7, 8	1, 2, 3, 4, 5	–

Gao et al., 2021			
- Risser classification	Risser stage 3, 4, 5	–	–
Hori et al., 2024			
- Sanders classification	Sanders stage 3A, 3B	–	–

Zapata et al., 2019			
- Risser classification	Risser stage 0	–	–

***Curve type***			

Schleder et al., 2023			
- Lenke classification	Lenke type 6	1, 3, 5	2, 4

Ylikoski, 2005			
- Curve morphology	All curve types (double, thoracic, thoracolumbar, lumbar)	–	–

Duval-Beaupere, 1996			
- Curve type	Thoraco-lumbar, lumbar	Thoracic, double-major, triple major	–

***Menarche status***			

Escalada et al., 2005			
- Menarche status	Every menarche status (from 2 years premenarchal to 5 years post menarche)		

***Growth velocity***			

Ylikoski, 2005 - Growth velocity (cm/year)	All growth velocities (<1, 1, 2, 3, 4, 5, ≥6)		

## 4 Discussion

With this systematic review we attempted to propose evidence-based radiological follow-up intervals for AIS patients, conservatively treated using observation alone, by categorizing patient- and radiological prognostic factors according to their corresponding monthly curve progression rate. In accordance with the previously stated aims of the current systematic review: (1) the rate of curve progression among the subgroups included within the individual studies (n = 9) ranged from non-significant to substantial monthly progression, indicating a broad spectrum of progression rates across subgroups; (2) four general prognostic factors with corresponding monthly progression rates were identified (bone age, curve type, menarche status and growth velocity), however; (3) no conclusive evidence was found to support the use of subsequent risk classification, based on aforementioned monthly curve progression rates in clinical practice. This was mainly due to the large heterogeneity observed within the included articles regarding the patient- and radiological characteristics that were studied for their rate of curve progression. Additionally, all the included studies were found to have a high risk of bias, mainly due to the conversion of patients with progressive curves to brace treatment. The natural curve progression could thereafter not be determined reliably due to the brace's moderating effect on deformity progression. The initiation of brace treatment therefore functioned as an endpoint for follow-up. Consequently, the average curve progression might be applicable for low-progressive curves, but the applicability for high-progressive curves is probably limited due to the exclusion of data that is captured during peak-height velocity. This bias is well observed within Table 4, as few studies report patient- and radiological characteristics at high risk for curve progression, and some studies only found curve progression rates that necessitate annual radiological follow-up and are thus at low risk for curve progression.

### 4.1 Bone age

In the literature, skeletal age has often been found to function as an important predictor for curve progression (Lenz et al., 2021; Sitoula et al., 2015). While curve progression slows at skeletal maturity, the growth spurt during adolescence (or peak height velocity), is a period where close monitoring of the deformity is necessary. Several radiological bone age classification systems were included in the current systematic review. The use of the Risser stage was most often observed (Cheung and Cheung, 2021; Gao et al., 2021; Zapata et al., 2019), followed by the DRU classification (Cheung and Cheung, 2021; Cheung et al., 2018) and the Sanders classification (Cheung and Cheung, 2021; Hori et al., 2024). Findings from the current study indicate certain Risser stage, radius-, ulna-, and Sanders-stages as medium-risk for curve progression (Table 4) which necessitates a corresponding radiological follow-up interval of six months. For the remaining skeletal ages that near skeletal maturity, annual follow-up should be sufficient. It is important to note that some inconsistency exists within the classification across the included studies (e.g. Sanders stage 3 is classified as low-risk in the study by Hori et al. (2024), while being moderate risk in the study by Cheung and Cheung (2021)). Additionally, the high risk of bias found within the included studies does not support the use of this risk classification in clinical practice, and the evidence remains inconclusive.

### 4.2 Curve type

The literature indicates that thoracic and double (thoracic) curves are associated with a higher risk of curve progression (Lenz et al., 2021; Wong et al., 2022). The current systematic review yielded similar results. Ylikoski (2005) described an annual progression rate of 2.4° and 2.1° respectively for thoracic and double curves, and a study by Duval-Beaupere (1996) noted an annual curve progression rate of 8.7°, 6.8°, and 9.1° for thoracic, double-major and triple-major curves respectively. However, some discrepancy exists between the two studies. The study byYlikoski (2005), indicates that thoracic and double curves have a higher progression rate compared to other curves, yet they are considered at low risk for curve progression according to current risk analysis. These results would suggest that annual radiological follow-up would be sufficient. However, the annual progression rates for thoracic, double-major and triple-major curves described by Duval-Beaupere et al. are categorized as moderate-risk curves, with a corresponding suggested radiological follow-up interval of six months. This discrepancy in curve progression rate might suggest the presence of some heterogeneity between both studies. While patient age at initial presentation (13.9 years vs 13.5 years) and Cobb angle at first appointment (23.4° vs 21.6°) were comparable, other factors as the inclusion of only girls in the study by Ylikoski (2005) might have caused this difference. The possible heterogeneity between the studies and discrepancy in results indicates that the risk classification by the current systematic review is not reliable for setting radiological follow-up intervals based on curve type.

Additionally, the study by Schleder et al. (2023) described curve progression rates according to the Lenke classification. The highest annual curve progression rates were observed in Lenke type 2 and 4, with average annual curve progressions of 11.43° and 34.84° respectively. However, this study included patients with an indication for the surgical correction of the deformity, and patients were observed in the period prior to surgery. Consequently, patients showed large initial magnitudes of the deformity at the start of the observation period (57.9 ± 14.67 (range 40–101)) and were therefore at higher risk for curve progression. These findings therefore cannot be used to state the frequency of radiological follow-up intervals among the general population of observed AIS patients, who generally present with smaller curves.

### 4.3 Menarche status

One study by Escalada et al. (2005) described curve progression rates according to the period before, or following menarche. It was found that closely before, during and closely after the menarche, the highest curve progression rates can be expected. Nevertheless, all periods before or after menarche with corresponding progression rates are still categorized as low-risk scoliosis in the current systematic review. According to these results, annual radiological follow-up would be sufficient. However, these results might be biased due to the conversion of observed patients to brace treatment. High progressive curves might subsequently not be considered in the radiological observation-only group. This suggestion is reinforced by the presence of high curve progression in the brace group, before menarche, after which the effect of brace treatment seems to stabilize curve progression. Therefore, the limited research and potential attrition bias complicate the use of menarche status to determine radiological follow-up intervals in current best-evidence synthesis.

### 4.4 Growth velocity

Among the included studies, Ylikoski (2005) conducted the only study that examined growth velocity (cm/year) in relation to annual curve progression. The highest annual curve progression occurs at a growth velocity of ≥6 cm/year (4.9°/year) and decreases with lower growth velocities. Yet, all are classified as low-risk curves according to our risk-categorization (annual radiological follow-up). As described earlier, the study by Ylikoski (2005) yielded lower curve progression rates compared to other included studies. Therefore, differences in study samples might be present. The level of evidence regarding growth velocity and annual curve progression consequently remains inconclusive.

### 4.5 Limitations

Several limitations of the current study must be acknowledged. In the first place, to establish recommendations for evidence-based radiological follow-up intervals, the inclusion criteria mandated a description of curve progression within a specific time frame (e.g. degrees/month). This allowed for the grouping of patient- or radiological characteristics in the risk categories of curve progression with corresponding recommendations for radiological follow-up intervals. However, this caused the exclusion of articles that did not note deformity progression in Cobb angle over a specific period, but some did however describe relevant trends. For example, a recent study by Yamamoto et al. (2020) stated recommendations for radiological follow-up based on the Cobb angle at presentation, and DRU grade. The high-risk group defined by this study (radiological follow-up intervals of <6 months) consisted of patients with radius grade 6 and 7, or ulnar grade 5. These findings show partial overlap with the current systematic review, with radius grades 6 and 7 deemed as moderate-risk (follow-up every 6 months), and ulnar grade 5 categorized as moderate- or high-risk (every 6 months or every 3–4 months follow-up respectively) depending on the study. Additionally, an initial Cobb angle of 30° or more is known to be significantly related to curve progression. A second shortcoming of the current systematic review therefore is the absence of studies stating (monthly) curve progression for already well-identified prognostic factors, such as curve magnitude at the first visit. Thus, no recommendations could be made regarding this radiological parameter and suggestions for radiological follow-up intervals. The initial Cobb angle has been proven to be strongly related to curve progression and should also be accounted for in clinical practice (Yamamoto et al., 2020; Lenz et al., 2021; Wong et al., 2022). At last, almost all studies made use of a retrospective study design, and associated biases might be present.

### 4.6 Recommendation

In the follow-up of conservatively treated AIS patients, it is important to limit the number of radiographs as much as possible, while recognizing curve progression promptly. Concerns regarding the cumulative number of radiographs have been raised in previous literature, given the generally young age of AIS patients and subsequent vulnerability to the carcinogenic effects of ionizing radiation (Law et al., 2016; Simony et al., 2016). In a study by Simony et al. (2016), AIS patients treated between 1983 and 1990 showed a 5 times higher cancer rate compared to an age-matched cohort, with an average of 16 radiographs conducted over the total treatment period. It is likely that modern radiography systems lead to a lower radiation exposure during follow-up, and that these risks therefore cannot directly be applied to patients currently undergoing observation. For example, modern techniques such as slit-beam imaging systems (EOS) and digital radiography substantially reduce radiation dose in full-spine imaging via improved detectors, child-specific exposure settings and optimized image processing (Cool et al., 2023; Rose et al., 2023; Ernst et al., 2018). In addition, a study by Heijboer et al. (2024) analyzed cancer prevalence rates among 337 female AIS patients treated between 1981 and 1995, and found no significant difference compared to the general population. Thus, there is no clear consensus in literature regarding the health impact of the frequent exposure of AIS patients to doses of ionizing radiation. However, according to the ALARA principle (As Low As Reasonably Achievable), an effort should still be made to reduce the radiation exposure of full-spine radiographs during follow-up of conservatively AIS patients. The current frequency of full-spine radiographs in conservatively treated AIS patients lacks a foundation in evidence-based literature. Thus, it is unknown whether it is justified to subject patients to such frequent full-spine radiographs. In this context, the current systematic review shows that there is a need for evidence to determine appropriate radiological follow-up intervals in conservatively treated AIS.

While important prognostic factors for curve progression in AIS have been well identified in currently available literature, they often do not incorporate the curve progression rate, with implications on follow-up (Lenz et al., 2021; Wong et al., 2022). This hampered the establishment of recommendations for radiological follow-up based on the pace of curve progression. The recent study by Yamamoto et al. (2020) formed recommendations for radiological follow-up based on the chance of detecting scoliosis progression within each month during a 1-year follow-up period. This study did not note the curve progression rate, but the percentage of progressive patients, therefore this study was excluded from the current systematic review. It is however, to our best knowledge, the only paper that focusses on the timing of radiological follow-up in observed AIS patients. Future studies should aim to establish clear recommendations for radiological follow-up based on already well identified clinically relevant prognostic factors for progression rate combined with clinical outcome. Additionally, the reporting of curve progression rates should be included in studies, in order to form evidence-based guidelines for radiological follow-up intervals. In clinical practice, radiological intervals should also be guided by clinical assessment. Findings upon examination, such as increasing angle of trunk rotation on scoliometer, or worsening trunk or shoulder asymmetry may justify prompt radiography, while clinical stability may support the postponement of imaging. This approach was also stated in the SOSORT consensus paper (Negrini et al., 2018). However, because these clinical indicators were not systematically reported in the included studies, they were not part of the current analysis and are mentioned here as practical consideration.

## 5 Conclusion

Based on results of the current systematic review, AIS patients who do not receive treatment except for radiological follow-up should be radiologically evaluated every six months until skeletal maturity is approached, after which a brief period of annual follow-up should be sufficient. However, the studies included showed a high risk of bias, as well as a substantial heterogeneity among the studied prognostic factors for curve progression. Additional studies are needed to investigate the curve progression rates related to the most important prognostic factors on the curve progression in AIS, which possibly leads to new recommendations regarding the radiological follow-up intervals.

## Author contribution

All authors contributed to the study conception and design. Material preparation, data collection and analysis were performed by JC, AMP, BJR, FSJ and JS. The first draft of the manuscript was written by JC, and all authors commented on previous versions of the manuscript. All authors read and approved the final version of the manuscript.

## Ethics approval

Ethical approval was not applicable given the study design (systematic review)

## Funding

This research did not receive any specific grant from funding agencies in the public, commercial, or not-for-profit sectors.

## Declaration of competing interest

The authors declare that they have no known competing financial interests or personal relationships that could have appeared to influence the work reported in this paper.

## Data Availability

The datasets generated during and/or analyzed during the current study are available from the corresponding author on reasonable request.

## Glossary

AIS: Adolescent Idiopathic Scoliosis
SRS: Scoliosis Research Society
PA: Posterior-Anterior
SSE: Scoliosis-Specific Exercise
SOSORT: The International Scientific Society on Scoliosis Orthopaedic and Rehabilitation Treatment
ACR: American College of Radiology
PRISMA: Preferred Reporting Items for Systematic Reviews and Meta-Analyses
QUIPS: Quality in Prognostic Studies
DRU: Distal Radius and Ulna
ALARA: As Low As Reasonably Achievable
