# Supplementary material for: Radiological follow-up strategies in adolescent idiopathic scoliosis patients: A best evidence synthesis by systematic review
Source: Brain Spine. 2025 Nov 5;5:105865. doi: 10.1016/j.bas.2025.105865 (PMC12663028; doi:10.1016/j.bas.2025.105865)
Supplement: Multimedia component 1 [file mmc1.docx]

MEDLINE (OVID):

Database(s): **Ovid MEDLINE(R) ALL**
Search Strategy:

| **#** | **Searches** |
| --- | --- |
| 1 | Scoliosis/ and Adolescent/ |
| 2 | (adolescen* adj3 idiopathic scoliosis).ti,ab,kf. |
| 3 | 1 or 2 |
| 4 | (Scoliosis/dg or Radiography/) and disease progression/ |
| 5 | ((scoliosis or curve or deformit*) adj3 progress*).ti,ab,kf. |
| 6 | 4 or 5 |
| 7 | follow-up studies/ or longitudinal studies/ or Outcome Assessment, Health Care/ |
| 8 | (follow up or followup or followed up or predict* or assessment).ti,ab,kf. |
| 9 | 7 or 8 |
| 10 | 3 and 6 and 9 |
| 11 | (exp Animals/ or exp Animal Experimentation/ or exp models, animal/ or (rat or rats or mice or mouse or murine or murines or rodent or rodents or rabbit or rabbits or cat or cats or dog or dogs or pig or pigs or cow or cows or monkey or monkeys or goat or goats or horse or horses or ape or apes or gorilla or gorillas or sheep or sheeps or ovine or lamb or swine or swines or porcine or pup or pups or canine or beagle).ti,ab,kf.) not Humans/ |
| 12 | 10 not 11 |
| 13 | letter/ or comment/ or editorial/ or (letter or comment* or editorial).ti. |
| 14 | 12 not 13 |

EMBASE OVID:

Database(s): **Embase Classic+Embase**
Search Strategy:

| **#** | **Searches** |
| --- | --- |
| 1 | adolescent idiopathic scoliosis/ |
| 2 | idiopathic scoliosis/ and adolescent/ |
| 3 | *scoliosis/di, dm, rt, th and adolescent/ |
| 4 | (adolescen* adj3 idiopathic scoliosis).ti,ab,kf. |
| 5 | 1 or 2 or 3 or 4 |
| 6 | (spine radiography/ or scoliosis/di or adolescent idiopathic scoliosis/di) and (disease course/ or disease severity/ or disease exacerbation/) |
| 7 | ((scoliosis or curve or deformit*) adj3 progress*).ti,ab,kf. |
| 8 | 6 or 7 |
| 9 | exp "evaluation and follow up"/ or follow up/ or prediction/ or long term care/ or outcome assessment/ or outcomes research/ or treatment outcome/ |
| 10 | (follow up or followup or followed up or predict* or assessment).ti,ab,kf. |
| 11 | 9 or 10 |
| 12 | 5 and 8 and 11 |
| 13 | (exp animal/ or exp animal experiment/ or exp experimental animal/ or exp animal model/ or nonhuman/ or (rat or rats or mice or mouse or murine or murines or rodent or rodents or rabbit or rabbits or cat or cats or dog or dogs or pig or pigs or cow or cows or monkey or monkeys or goat or goats or horse or horses or ape or apes or gorilla or gorillas or sheep or sheeps or ovine or lamb or swine or swines or porcine or pup or pups or canine or beagle).ti,ab,kw.) not human/ |
| 14 | 12 not 13 |
| 15 | letter/ or editorial/ or note/ or (letter or comment* or editorial).ti. |
| 16 | 14 not 15 |
| 17 | limit 16 to conference abstracts |
| 18 | 16 not 17 |

[Cochrane Database of Systematic Reviews](https://www.cochranelibrary.com/)

Issue 1 of 12, January 2025

[Cochrane Central Register of Controlled Trials](https://www.cochranelibrary.com/)

Issue 12 of 12, December 2024

ID Search Hits

#1 (adolescent idiopathic scoliosis):ti,ab,kw

#2 (adolescen* and scoliosis):ti,ab,kw

#3 #1 or #2

#4 MeSH descriptor: [Scoliosis] explode all trees and with qualifier(s): [diagnostic imaging - DG] 109

#5 ((scoliosis or curve or deformit*) near/3 progress*):ti,ab,kw

#6 #4 or #5

#7 (follow up or followup or followed up or predict* or assessment):ti,ab,kw
